# Spiral groove bearing design for improving plasma skimming in rotary blood pumps

**DOI:** 10.1007/s10047-023-01422-y

**Published:** 2023-12-28

**Authors:** Ming Jiang, Wataru Hijikata

**Affiliations:** Department of Mechanical Engineering, Tokyo Institute of Technology, 2-12-1 Ookayama, Meguro-Ku, Tokyo, 152-8550 Japan

**Keywords:** Hydrodynamic bearing, Optimal design, Plasma skimming, Rotary blood pumps, Spiral groove bearing

## Abstract

High-efficiency plasma skimming is hopeful to prevent hemolysis inside spiral groove bearings (SGBs) because it can exclude red blood cells from the ridge gap with a high shear force. However, no study reveals the shape design of SGBs to improve plasma skimming. Therefore, this study proposed and applied a groove design strategy to designing an optimal SGB for enhancing plasma skimming in a rotary blood pump (RBP). Initially, we proposed the design strategy that the shape of the groove for enhancing plasma skimming corresponds to the direction of blood flow in the ridge gap. Second, we visualized the cell flow in a specially designed experimental RBP to determine the direction of blood flow, which was helpful in the subsequent SGB design. Then, we created an SGB to provide superior plasma skimming and applied it to the experimental RBP. We evaluated the plasma skimming effect of SGB at rotational speeds ranging from 2400 to 3000 rpm and hematocrit conditions between 1% and 40%. At a 1% hematocrit, the plasma skimming efficiency for the entire SGB was greater than 95%. In all hematocrit conditions, the efficiency at the inner ridges of the SGB was greater than 80%. The results showed the designed SGB successfully induced excellent plasma skimming within ridge gaps. This study is the first to propose and apply a shape design strategy to generate excellent plasma skimming within an SGB. This study may contribute to the prevention of SGB hemolysis inside SGB for use in RBPs.

## Introduction

The use of hydrodynamic bearings inside rotary blood pumps (RBPs) has been extended owing to their simplicity and durability [1, 2]. Spiral groove bearings (SGBs), a type of hydrodynamic bearing, are small and have a high load-carrying capacity (LCC) [3]. Various studies have targeted enhancing the hydraulic performance [4–6] and hemocompatibility [7, 8] of SGBs through innovative structural designs. However, the risk of hemolysis induced by high shear stress in small bearing gaps remains an obstacle to the wider application of SGBs [9].

A common approach to mitigate hemolysis is to increase the bearing gap. While this reduces the hemolysis risk, it also decreases the LCC of the SGB, often necessitating structural modifications to compensate. Murashige et al. improved the LCC of an SGB to increase the bearing gap size from 25 to 88 μm by creating a width-decreasing groove in the blood flow direction [8]. Yamane et al. obtained total bearing gap sizes ranging from 25 to 63 μm by rearranging the groove and ridge areas on the SGB [10]. Nonetheless, the pump’s volume and the performance constraints of the LCC limit the extent to which the bearing gap can be increased, leaving hemolysis in SGBs unsatisfactory [11].

Thus, another potential solution was considered, inspired by plasma skimming between the ridge and groove gaps within the SGB. Plasma skimming denotes the hematocrit reduction inside the ridge gap relative to the initial hematocrit within the pump. If the hematocrit in the ridge gap could be minimized, red blood cells would be excluded from the ridge gap, thereby preventing hemolysis caused by high shear stress. Therefore, as the first step, the goal is to improve plasma skimming and decrease the hematocrit in the ridge gap.

Despite this potential solution, no studies have focused on designing the shape of SGBs to enhance plasma skimming. Existing studies have documented the occurrence of plasma skimming in SGBs and evaluated the hematocrit within the ridge gap [12–15].

This study aimed to address this gap by proposing a specially designed SGB to improve plasma skimming in the ridge gap. First, we proposed a strategy that the groove shape should align with blood flow within the ridge gap to improve plasma skimming, building on our previous experimental findings [16]. Next, utilizing a genetic algorithm (GA) and numerical analysis of the generated LCC, we created an optimal SGB supposed to induce high-efficiency plasma skimming while maintaining sufficient LCC. Finally, we implemented this optimal SGB in a specially designed experimental RBP and evaluated its plasma skimming performance.

## Materials and methods

### Shape design strategy to enhance plasma skimming

The shape design, derived from our previous work [16], demonstrates that an excellent plasma skimming effect occurs when the angle between the blood flow and the groove’s tangent is less than 5 degrees. Thus, the following design strategy was established: The groove shape of the SGB should be aligned with the blood flow direction within the ridge gap to enhance plasma skimming. This groove shape was generated based on the path line of a hypothetical particle in the defined planar velocity field. To ascertain the blood flow direction within the ridge gap, we conducted measurements using a specially designed experimental RBP.

### Generating SGB geometry

**Fig.1:**
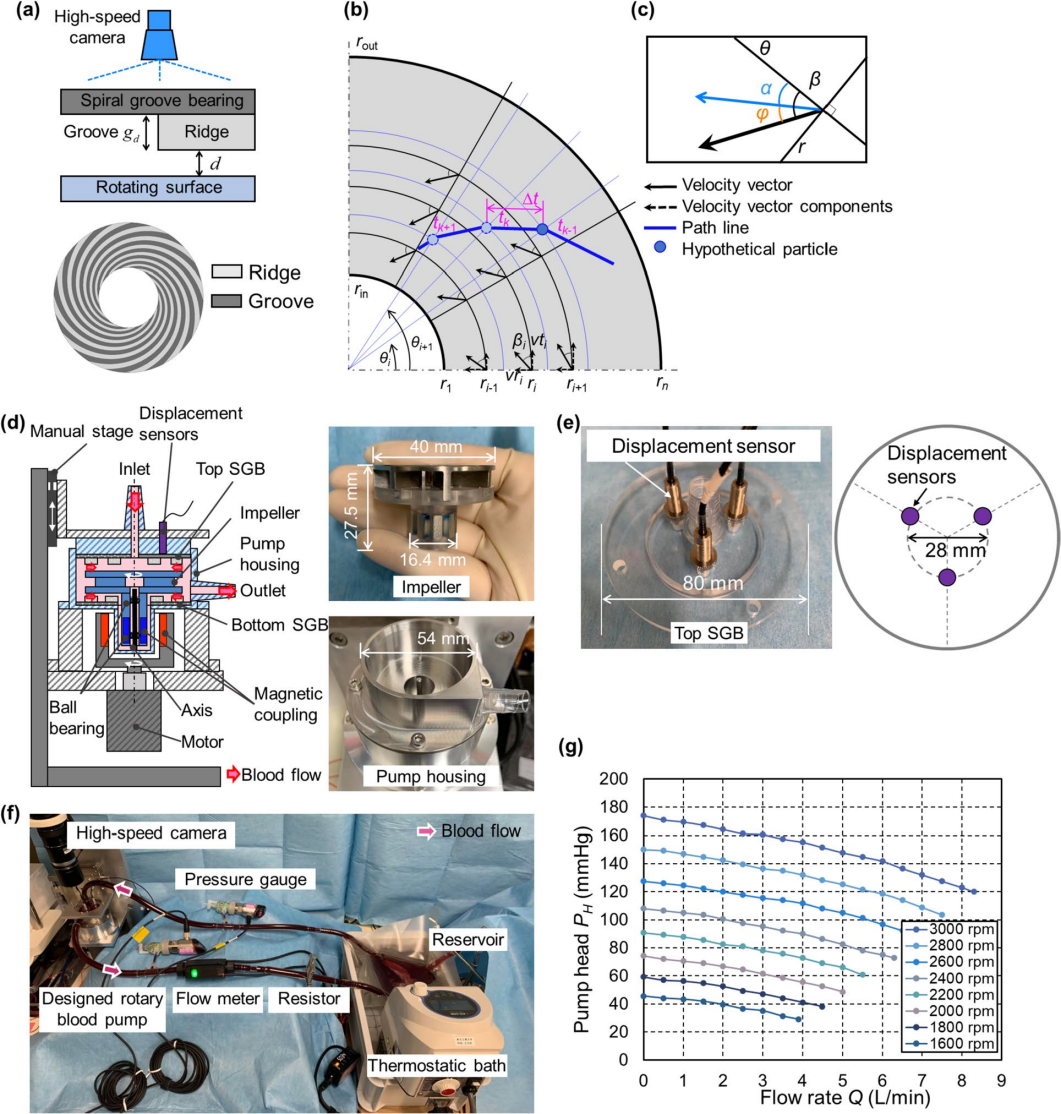
**a** Structure and cross-sectional view of grooves and ridges in SGBs. **b** Spiral groove shape, which is drawn following the path line of a hypothetical particle. **c** The relationship among the angles *β*, *α*, and *φ*. **d** Structure of the specifically designed experimental RBP and the photographs of the impeller and pump housing. **e** Three displacement sensors embedded on the top SGB. **f** The blood circuit for both measuring the blood flow inside the ridge gap and evaluating plasma skimming effect. **g** Pump performance of the designed experimental RBP

Figure 1(a) illustrates the structure of SGBs, where *d* represents the ridge gap size and *g*_*d*_ is the groove depth. Figure 1(b) depicts the groove shape of SGBs, created by generating a path line for a hypothetical particle in a defined velocity field. First, the region extending from the SGB’s inner radius *r*_in_, to the outer radius *r*_out_, is evenly divided into *n*-1 sections by *n* nodes in a radial arrangement. The variables *r* and *θ* represents the radial and circumferential coordinate, respectively. The *i-*th vector in the velocity field is characterized by the circumferential and radial components *vt*_*i*_ and *vr*_*i*_, respectively. The angle *β*_*i*_ of the vectors relative to the circumferential direction is given by Eq. (1), and *vt*_*i*_ has a linear relationship with *r*_*i*_, as shown in Eq. (2), where the constant *c* equals 1.

1 $$ vr_i = vt_i \ \tan \beta_i $$

2 $$ vt_i = c\ r_i $$

Next, a hypothetical particle is released into the velocity field along *r*_out_, and its path is traced by connecting its positions at different times *t*_*k*_ using a sampling time ∆*t*. Interpolating the vectors at neighboring nodes provides the local velocity vectors for the hypothetical particle at each moment. Since *vt*_*i*_ is defined by Eq. (2), varying *β*_*i*_ produces different vector fields, each of which defines a spiral groove shape. The value of *β* is defined as the sum of two angles, as expressed in the following equation and illustrated in Fig. 1(c).

3 $$ \beta = \alpha + \varphi $$

The spiral groove design process includes measuring the blood flow direction *α*, which denotes the angle of the blood flow relative to the circumferential direction, and designing an appropriate *φ*, which is the angle between the direction of blood flow and the vector of the hypothetical particle.

### Experimental setting

This section outlines the experimental setup used to both measure blood flow and evaluate plasma skimming. Figure 1(d) illustrates the structure of the experimental RBP. The impeller, equipped with two ball bearings, is designed to slide smoothly along the axis. Two identical SGBs at the top and bottom side of the impeller, hydrodynamically levitate the impeller in the thrust direction. The impeller has a weight of 40 g, and the magnetic force exerted on it by the magnetic coupling is designed to be 0.9 N in the upward direction. Consequently, the conservative and static estimation for the required LCC is the combined value of gravitational and magnetic forces, totaling 1.3 N.

The top SGB is connected to a manual stage (BSS16-100C; SURUGA SEIKI CO., LTD., Shizuoka, Japan), allowing adjustments to the bearing gap during the experiment. The ridge gap size, formed by the top SGB and the impeller surface, is monitored and analyzed using three eddy current displacement sensors (PU-05; Applied Electronics CO., LTD., Kanagawa, Japan), as depicted in Fig. 1(e). All the SGBs used in this study were manufactured by a professional resin manufacturer (ABMODEL CO., LTD., Kanagawa, Japan).

Fresh porcine blood, with hematocrit adjusted using phosphate-buffered saline (FUJIFILM, Wako Pure Chemical CO., LTD., Osaka, Japan), is circulated within the circuit shown in Fig. 1(f). The pump flow rate is maintained at 5 L/min during experiments, using a flow meter (FD-XS20; Keyence CO., LTD., Osaka, Japan) and a resistor (MonotaRO CO., LTD., Hyogo, Japan). The pressure is monitored by a pressure gauge (GP-M001; Keyence), and the experiment is conducted at a controlled temperature of 37 °C, maintained with a thermostatic bath (TM-3; AS ONE CO., Ltd., Osaka, Japan). Figure 1(g) details the RBP performance under this experimental setting. During the experiments, the blood flow between the top SGB and impeller surface is visualized using a high-speed camera (VW-9000; Keyence) set at a frame rate of 4000 fps.

### Measuring blood flow

Due to the high shear rate, the Couette flow, induced by the rotating impeller, dominates the blood flow within the ridge gap. When the ridge gap is set to 20 μm and the impeller’s rotational speed ranges from 2400 to 3000 rpm in this study, the SGB’s groove shape minimally influences the Couette blood flow direction within the ridge gap.

**Fig. 2:**
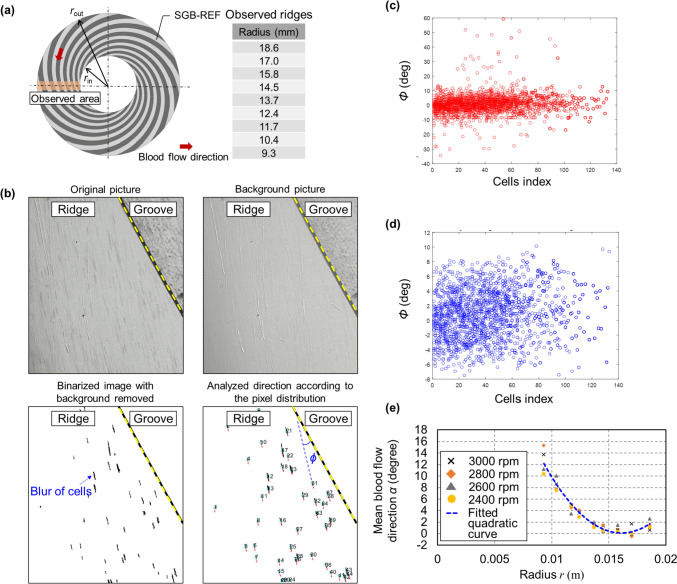
**a** The observed area on SGB-REF at different radii for measuring the direction of blood flow. **b** Examples of analyzing blood flow direction *ϕ* based on one frame of photographed cells’ blur at the radius of 18.6 mm and a rotational speed condition of 3000 rpm. **c** Plotted blood flow direction relative to the tangent of the ridge for 20 frames in a single rotation of the impeller. **d** Data within the one-sigma Gaussian distribution derived from the data in (**c**). **e** The measured mean blood flow direction of the data in (**d**) at different radii with the rotational speed range of 2400 to 3000 rpm, and the fitted curve of the flow direction relative to different radii

Consequently, an SGB-REF, as depicted in Fig. 2(a)**,** was employed to visualize blood flow within the ridge gap. The SGB-REF’s spiral curve, defined by the following equation, consists of 12 grooves and 12 ridges having equal width.

4 $$ r = r_{in} e^{\theta \tan 16^{\circ} } $$

The blood flow direction was observed and analyzed at 2400, 2600, 2800, and 3000 rpm, respectively. The hematocrit condition was set to 0.1% to capture a clear image of the blood flow, thereby preventing RBC blur from overlapping. The shutter speed of the high-speed camera was set to 1/50000 s.

Analysis of the blood flow direction was conducted based on video footage. Figure 2(b) illustrates the example process of analyzing one single frame. After extracting the background from the photographed blood cells, the images of the blurry RBCs were converted into black pixels on a binarized image. The blood flow direction was estimated by approximating the blur distribution as a straight line and measuring the angle *ϕ* between the line and the tangent of the groove edge. Since SGB-REF has a fixed 16° angle between the groove tangent and the circumferential direction, the blood flow direction (defined as the angle of the blood flow direction relative to the circumferential direction) can be calculated based on *ϕ* using the following equation:

5 $$ \alpha = 16 - \phi . $$

Figure 2(c) illustrates the analyzed *ϕ* across 20 frames during a single rotation of the impeller. To mitigate the influence of measurement inaccuracies, the data in Fig. 2(c) were subjected to Gaussian distribution analysis. The data represented in Fig. 2(d), which are within one standard deviation (one-sigma) of the values in Fig. 2(c), was used to calculate the mean *ϕ*.

Figure 2(e) summarizes the mean analyzed angle of all photographed blurs relative to the SGB radii at different rotational speed conditions. To estimate *α* at any *r* value, the data obtained from the four rotational speed conditions were fitted to one quadratic curve using the following equation, as shown in Fig. 2(e). Figure 2(e) implies that there is no significant difference in the blood flow direction across 2400–3000 rpm.

6 $$ \alpha = 260495r^2 - 8394.3r + 67.7 $$

### Optimization of the SGB shape

After measuring *α*, the subsequent step is to design an appropriate *φ* for creating the SGB shape. The goal of this study is to induce an excellent plasma skimming effect while ensuring the SGB provides sufficient LCC to levitate the impeller. Additionally, from a physiological perspective, a smooth groove shape is desired to prevent abrupt changes in the flow path. To meet these objectives, GA was employed to optimize the SGB shape, and the Reynolds equation was used for the numerical analysis of the SGB’s LCC.

The objective function *I* for optimizing the SGB shape is defined as**:**

7 $$ \begin{aligned} \hfill \min \;I(\varphi ) & = w_p \ \frac{1}{\varphi_0^2 }\ \ \frac{1}{n}\ \sum_{i = 1}^n {\varphi_i^2 } + w_f \ \frac{1}{f_0^2 }(f_0 - f(\varphi ))^2 \nonumber \\ & \quad + w_g \ \frac{1}{\varphi_d^2 }\frac{1}{n - 1}\sum_{i = 1}^{n - 1} {(\frac{{\varphi_{i + 1} - \varphi_i }}{\Delta r})^2 } \nonumber \\ & \quad  subject\;to \, \quad 2 < \varphi < 5, \\ \end{aligned} $$

where ***φ*** is the vector of design variable *φ*_*i*_ (*i* = 1, 2,.., n) defined in Eq. (3) to produce the velocity vector field for the hypothetical particle. The coefficients *w*_*p*_,* w*_*f*_*,*_*,*_ and *w*_*g*_, and the constants *φ*_0_, *f*_0_, and* φ*_*d*_ serve for weighting and normalizing the terms. The equation’s first term characterizes the difference between the spiral groove design and the measured blood flow direction. The second term calculated the *f*(***φ***), representing the LCC of the generated SGB shape concerning ***φ***, with a negative sign since a higher LCC is preferable. By computing the difference between two adjacent variable elements relative to the interval ∆*r*, the third term aims to create a smooth shape for the SGB along the radial axis.

**Fig. 3:**
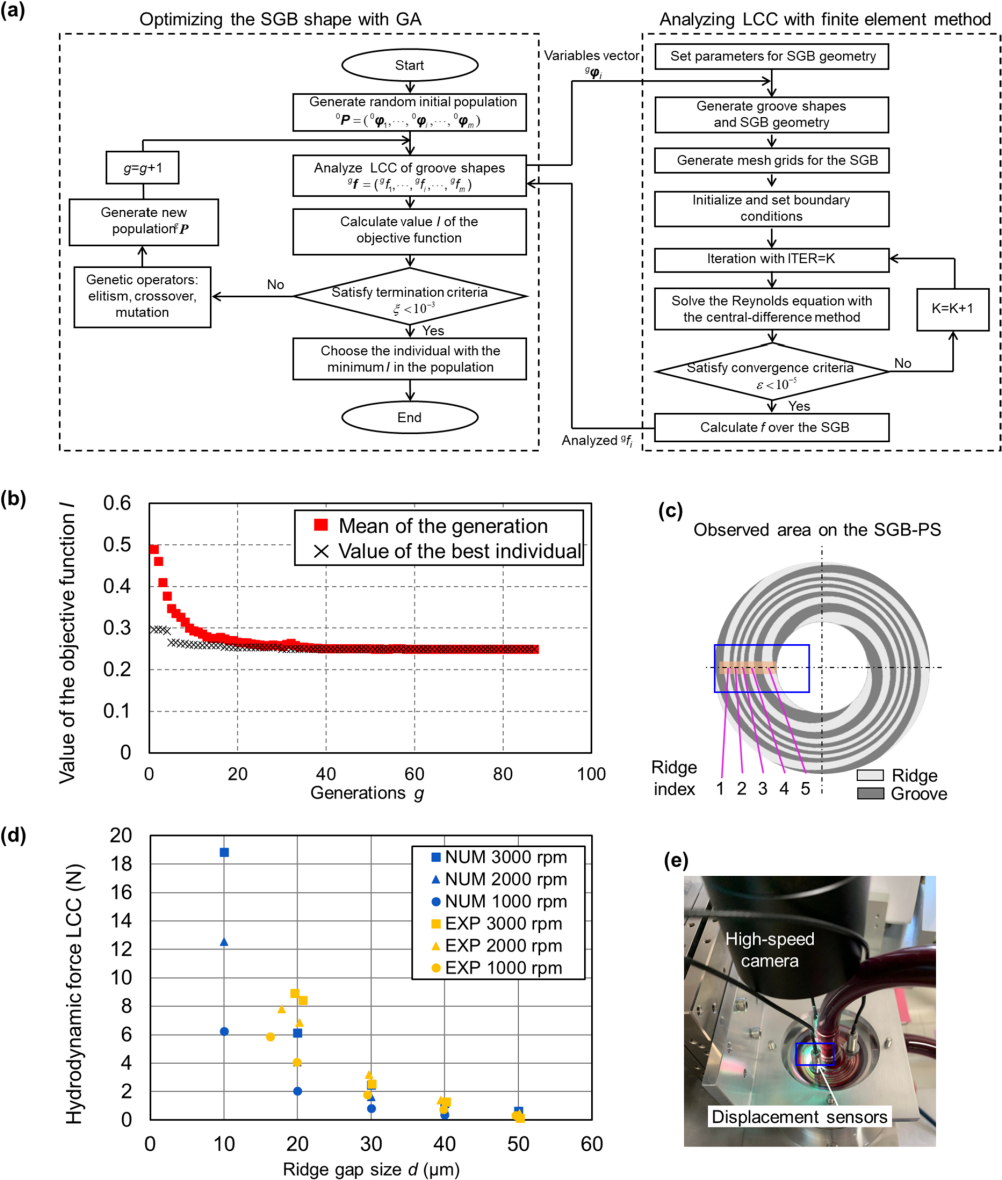
**a** Flowchart for optimizing the SGB shape using GA combined with the numerical LCC analysis. **b** Measured (EXP) and the numerically analyzed (NUM) LCC of SGB-PS. Numerical analysis condition: Inlet pressure *p*_*in*_ = 0, outlet pressure *p*_*out*_ = 0 and viscosity *μ* = 3.1 mPa·s with the glycerin solution. **c** Values of the objective function in the iterative computation of the optimization process. **d** The created SGB-PS and the observed ridges area on it during the experiment for evaluating plasma skimming effect. **e** Experimental view of the observed SGB-PS during the experiment

The optimization process is illustrated in the flowchart in Fig. 3(a) with the population *P* containing *m* individuals, and *g* representing the current generation. The GA process generates a new generation ^*g*+1^*P* based on the existing generation ^*g*^*P* and the calculated *I*, continuing until no improvement in the mean *I* is observed between generations.

The function *f*(***φ***) can be analyzed based on the 2D Reynolds Eq. (8) and (9) to calculate the generated pressure in cylinder coordinates [17]. The values of the parameters and the boundary conditions are summarized in Table 1(a).

8 $$ \frac{\partial }{\partial r}(H^3 r\frac{\partial p}{{\partial r}}) + \frac{1}{r}\frac{\partial }{\partial \theta }(H^3 \frac{\partial p}{{\partial \theta }}) = 6\mu \omega r\frac{\partial H}{{\partial \theta }} $$

**Table 1:** Optimized value of the variables ***φ*** for generating SGB-PS

Variables	Value (degree)	Variables	Value (degree)
*φ*_*1*_	2.0004	*φ*_*11*_	2.0000
*φ*_*2*_	2.0000	*φ*_*12*_	2.0624
*φ*_*3*_	2.0000	*φ*_*13*_	2.0155
*φ*_*4*_	2.0000	*φ*_*14*_	2.0000
*φ*_*5*_	2.0000	*φ*_*15*_	2.0000
*φ*_*6*_	2.0000	*φ*_*16*_	2.0018
*φ*_*7*_	2.0008	*φ*_*17*_	3.6290
*φ*_*8*_	2.0000	*φ*_*18*_	2.0187
*φ*_*9*_	2.7025	*φ*_*19*_	2.0000
*φ*_*10*_	2.0000	*φ*_*20*_	2.0000

*H* is the fluid film thickness equals ridge gap size *d*_*s*_ or *d*_*s*_ + *g*_*d*_ and *p* represents the local pressure. In the numerical analysis, the pressure *p*_*in*_ at *r*_*in*_, and *p*_*out*_ at *r*_*out*_ are set to zero. *μ* is the lubricant viscosity, and *ω* is the angular velocity of the impeller. *f* is obtained by integrating *p* over the SGB:

9 $$ f = \int_{r_{in} }^{r_{out} } {\int_0^{2\pi } {p\ r\ } } d\theta \ dr $$

The optimization procedure using GA encompasses the evaluation of *f*(***φ***) for each SGB shape as follows: The spiral curve was initially generated using ***φ*** as shown in Fig. 1(b), replicated to form the SGB geometry comprising three equal-width ridges and grooves. In this geometry, mesh grids were generated, and initial conditions were assigned for the coefficients of *p*_*in*_, *p*_out_, *d*_*s*_, *g*_*d*_, *μ,* and *ω.* Pressure values at each node were iteratively computed until satisfying the residuals criterion, and the evaluated *f* was passed to the GA. The optimization procedure was programmed and executed in MATLAB (MathWorks, U.S.A.).

The mean and minimum values of *I* for each generation are displayed in Fig. 3(b). The optimization terminates at the 87th generation. The optimized ***φ*** values are detailed in Table 1. Utilizing these optimized variables, the groove shape illustrated in Fig. 3(c) was created and labeled as SGB-PS. The LCC analysis based on the Reynolds equation was validated by measuring the LCC with the SGB-PS. Results presented in Fig. 3(d) show a consistent trend between the analyzed and measured LCC. When in the experimental condition with *d* less than 30 μm and rotational speed higher than 2000 rpm in this study, the measured LCC is larger than the required 1.3 N.

### Evaluating plasma skimming with the optimal SGB

Using the same experimental RBP and circuit as illustrated in Fig. 2(f), the plasma skimming effect of the SGB-PS was evaluated. This evaluation was conducted at rotational speeds of 2400, 2700, and 3000 rpm, and with hematocrit conditions ranging from 1% to 40%. Figure 3(c) and 3(e) provides a visual depiction of the observed ridges on SGB-PS, which were numbered from 1 to 5 for convenient indexing. The shutter speed of the high-speed camera was set to 1/900000 s.

Plasma skimming efficiency was analyzed based on the hematocrit within the ridge gap of the SGB-PS [14]. The plasma skimming efficiency, *E*, is defined by the following equation:

10 $$ E = \left(1 - \frac{Hct_r }{{Hct_c }}\right) \times 100\% , $$

*Hct*_*r*_ and *Hct*_*c*_ represent the hematocrits inside the ridge gap and circuit, respectively. *Hct*_*r*_ was derived using the following equation:

11 $$ Hct_r = \frac{q \times MCV}{{d \times \sigma_{RBC} }}, $$

*q* denotes the occupancy ratio of the RBCs in the photographed image, which was analyzed by counting the black pixels that represent the RBCs in a binarized picture [15]. The mean corpuscular volume *MCV* and cross-sectional area of RBCs *σ*_*RBC*_ are related by the following equation with *c*_*0*_ equals 0.39. *MCV* is measured with an automated hematology analyzer (MEK-6550; Nihon Kohden Corp., Tokyo).

12 $$ \sigma_{RBC} = \pi \left( {\frac{3MCV}{{4\pi c_0 }}} \right)^\frac{2}{3} $$

## Results

**Fig. 4:**
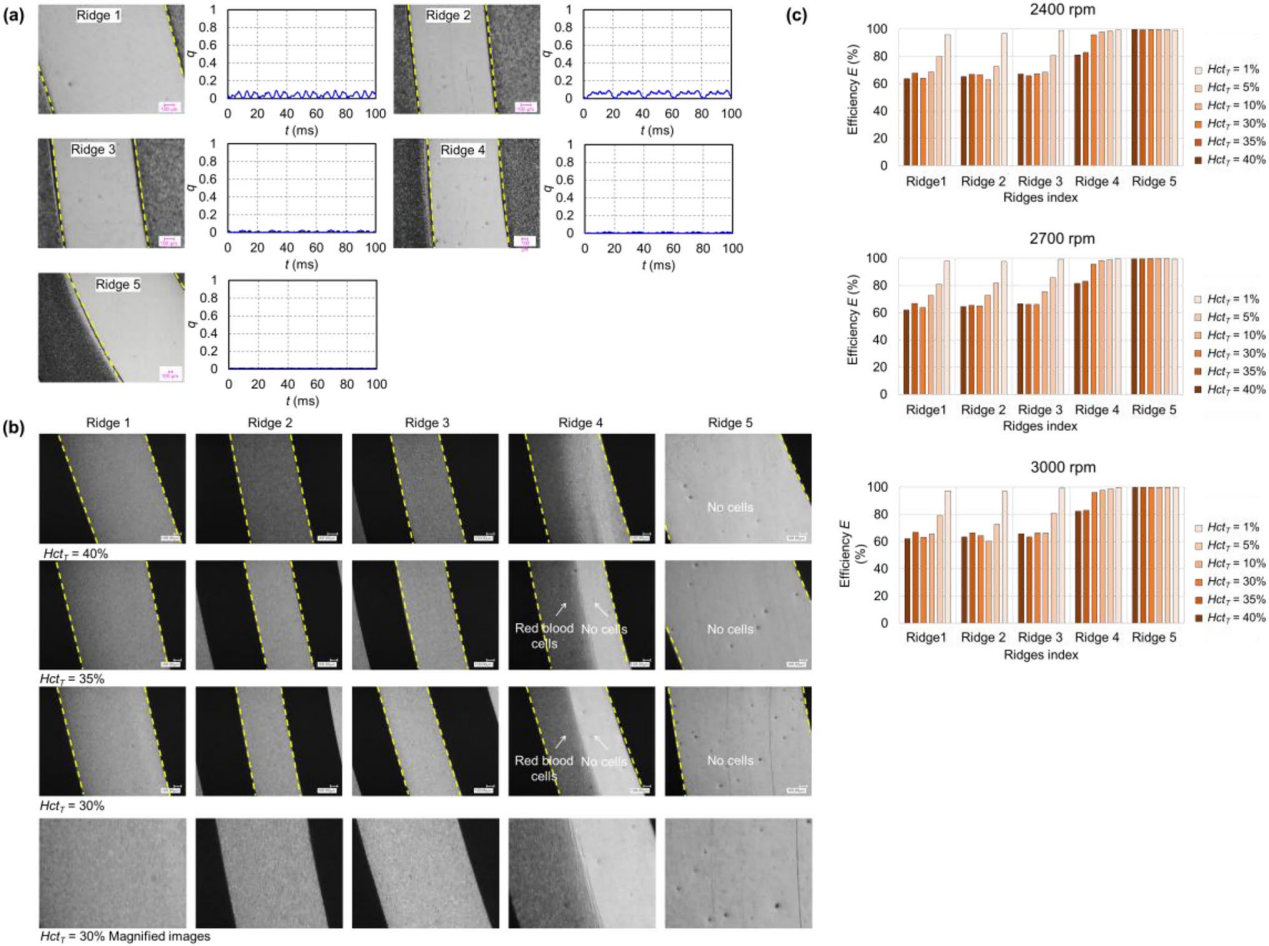
**a** Typical examples of photographed plasma skimming occurring inside ridge gaps of the SGB-PS with a hematocrit condition of 1% and a rotational speed of 3000 rpm at ridges from 1 to 5. The occupancy ratio of RBCs was plotted in a period of 100 ms. **b** Typical photographed plasma skimming occurring inside the SGB-PS under hematocrit conditions of 40%, 35%, 30% and the magnified images with hematocrit conditions of 30% for rotational speed of 3000 rpm. **c** Plasma skimming efficiency at varying hematocrit conditions for rotational speeds of 2400 rpm, 2700 rpm, and 3000 rpm

The coefficients and constants used in this study are specified in Table 2. The SGB-PS exhibited an outstanding plasma skimming performance, effectively excluding RBCs from the ridges. Figure 4(a) displays typical plasma skimming images and the derived occupancy ratio for 100 ms at each observed ridge, with a hematocrit of 1% and a rotational speed of 3000 rpm. In particular, ridges 3, 4, and 5 had an occupancy ratio close to zero, indicating remarkable plasma skimming within the ridge gaps. Ridges 1 and 2 had an occupancy ratio below 0.1.

**Table 2:** Values of the parameters and constants used for GA and numerical analysis

Variables (unit)	Value	Variables (unit)	Value
*r*_in_ (m)	0.008	*f*_*0*_ (N)	20
*r*_out_ (m)	0.02	*φ*_*d*_ (degree/m)	4.76 × 10^3^
*n*	20	*m*	40
*∆t* (s)	0.06	*d*_*s*_ (m)	2.0 × 10^–5^
*w*_*p*_	0.7	*g*_*d*_ (m)	1.0 × 10^–4^
*w*_*f*_	0.2	*μ* (mPa·s)	3
*w*_*g*_	0.1	*ω* (rad/s)	251.3
*∆r* (m)	6.3 × 10^–4^	*p*_in_ (Pa)	0
*φ*_*0*_ (degree)	5	*p*_out_ (Pa)	0

With hematocrit levels reaching up to 40%, the SGB-PS effectively induced plasma skimming on the inner side of the bearing. Figure 4(b) shows the images of plasma skimming at hematocrit conditions of 40%, 35%, and 30%, along with magnified images at hematocrit of 30%, all taken at a rotational speed of 3000 rpm. The RBCs in the gap of ridge 5 were effectively excluded. The plasma skimming within ridges 5 and 4 is superior compared to the other three ridges.

Figure 4(c) illustrates the plasma skimming efficiency for various ridges under different rotational speeds and hematocrit conditions. Repeated experiments for each condition are noted in Table 3**.** *HCT*_*T*_ represents the target hematocrit for each test. Overall, ridges 5 and 4 maintained efficiencies of over 80% for all hematocrit conditions. The average ridge gap sizes varied between 17 and 23 μm under different hematocrit and rotational speed conditions. Table 4 lists the measured hematocrits in the circuit for different conditions.

**Table 3:** The numbers of experiments conducted in each condition for evaluating plasma skimming effect

	*Hct*_*T*_ = 1%			*Hct*_*T*_ = 5%			*Hct*_*T*_ = 10%		
	2400 rpm	2700 rpm	3000 rpm	2400 rpm	2700 rpm	3000 rpm	2400 rpm	2700 rpm	3000 rpm
Ridge 1	4	4	4	4	3	4	5	4	5
Ridge 2	4	4	4	4	3	4	5	4	5
Ridge 3	4	4	4	4	3	4	5	4	5
Ridge 4	4	4	4	4	3	4	5	4	5
Ridge 5	4	4	4	4	3	4	5	4	5
	*Hct*_*T*_ = 30%			*Hct*_*T*_ = 35%			*Hct*_*T*_ = 40%		
	2400 rpm	2700 rpm	3000 rpm	2400 rpm	2700 rpm	3000 rpm	2400 rpm	2700 rpm	3000 rpm
Ridge 1	4	4	4	4	4	4	4	4	4
Ridge 2	4	4	4	4	4	4	4	4	4
Ridge 3	4	4	4	4	4	4	4	4	4
Ridge 4	4	4	4	4	4	4	4	4	4
Ridge 5	4	4	4	4	4	4	4	4	4

**Table 4:** Measured mean circuit hematocrit *HCT*_*c*_ at different experimental conditions

Measured hematocrit *Hct*_*c*_	*Hct*_*T*_	Test 1			Test 2	Test 3	Test 4	Test 5
	1%	1.1 ± 0.0			1.0 ± 0.0		0.7 ± 0.0	1.5 ± 0.0
	5%	5.4 ± 0.1			4.3 ± 0.0		3.5 ± 0.0	4.6 ± 0.1
	10%	8.0 ± 0.1			9.0 ± 0.1	9.4 ± 0.2	7.9 ± 0.1	7.3 ± 0.2
	30%				29.1 ± 0.3	28.6 ± 0.1	28.2 ± 0.1	31.6 ± 0.2
	35%				35.1 ± 0.2	33.5 ± 0.3	34.3 ± 0.1	34.1 ± 0.3
	40%				39.7 ± 0.3	39.0 ± 0.2	39.8 ± 0.4	39.7 ± 0.2
Measured *MCV*								
fL	1%	56.9 ± 1.4			54.6 ± 1.4		60.1 ± 2.5	60.0 ± 0.0
	5%	58.2 ± 0.4			52.7 ± 0.6		57.2 ± 0.3	59.0 ± 0.2
	10%	59.0 ± 0.5			53.0 ± 0.4	54.5 ± 0.2	57.8 ± 0.3	59.9 ± 0.6
	30%				53.4 ± 0.3	52.8 ± 0.2	57.0 ± 0.2	59.4 ± 0.3
	35%				52.8 ± 0.3	53.3 ± 0.4	57.3 ± 0.4	59.2 ± 0.4
	40%				53.0 ± 0.1	53.8 ± 0.4	57.2 ± 0.3	59.3 ± 0.3

## Discussion

This study proposes an approach to enhance plasma skimming within the SGB ridge gap by modifying the groove shape. For the first time, the SGB-PS achieves excellent plasma skimming across radial ridges. Previous studies on plasma skimming in the ridge gap of SGB have primarily focused on plasma skimming on a specific radius [14, 15, 17]. In this study, the overall plasma skimming effect was considered in the SGB design by measuring the blood flow direction in different radii.

Compared to previous studies, the SGB-PS demonstrated superior plasma skimming effects in ridge gaps. Murashige et al. reported a peak plasma skimming efficiency of 93% at a 21-μm ridge gap size and 78% at a 23-μm ridge gap size, given a 1% hematocrit and 3000 rpm rotational speed condition [14]. While Fig. 4(c) demonstrates that the SGB-PS achieves an efficiency exceeding 95% across all conditions. Sakota et al. [15], utilizing the same RBP as [14], observed a plasma skimming efficiency with hematocrit up to 35%, where the occupancy ratio was close to 1. In our study under the same hematocrit, RBCs were effectively excluded from ridges 4 and 5, with efficiencies surpassing 82% and 99%, respectively. The SGB-PS also implies a better plasma skimming effect than SGB-REF. Because the SGB-REF retained RBCs in the ridge gap for flow direction analysis at a 0.1% hematocrit, the SGB-PS nearly eliminated all cells even at a higher hematocrit of 1%.

In our investigation, ridges 4 and 5 on the SGB-PS exhibited a more effective plasma skimming performance compared than the outer side ridges. Referencing Table 1, the radii for *φ*_*8*_* and φ*_*9*_ are noted as 12.4 mm and 13.1 mm, respectively. In Fig. 4(b)**,** there is a boundary of plasma skimming on ridge 4. The observed ridge 4 is at the radii ranging from 12.6 mm to 13.3 mm. Given this correlation in radii, one possible reason is considered that the optimized parameter for the inner ridges of the SGB-PS is the lower limit of 2 degrees, which contributes to the plasma skimming effect. On the outer side, there are optimal variables of which the value is 2.7 and 3.6 degrees, which contributes to the LCC more. As a result, the inner groove shape of SGB-PS is close to the direction of blood flow, possibly inducing remarkable plasma skimming. These findings necessitate in-depth research and substantial supporting evidence in the future.

Additionally, the insights derived from our validated design strategy may suggest the mechanism of plasma skimming within the SGBs. Plasma skimming within SGBs is thought to prevent RBCs from entering the ridge gap, and the lift force keeps RBCs within the groove. The dominant Couette flow in the bearing gap has a mixing effect on the flow of grooves and ridges and transports RBCs from the groove gap to the ridge gap. Therefore, the SGB groove must correspond to the blood flow within the ridge gap to prevent RBCs from entering. Assuming that the groove is identical to the blood flow, the RBCs that entered the ridge initially would not be able to leave. Therefore, it is believed that a lift force must act on RBCs to prevent them from migrating from the ridge gap to the groove gap. This lift force is most likely the result of the perpendicular velocity gradient between the groove and ridge.

Besides, the higher plasma skimming efficiency was observed with lower hematocrit conditions as shown in Fig. 4(c). Lower hematocrit means lower volume ratio of red blood cells in the circuit. Therefore, there is a lower possibility of mixing the red blood cells from the groove to the ridge area compared with high hematocrit condition. Future research interests on investigating the hematocrit in groove area may provide better understanding of plasmas skimming mechanism.

Though the findings provide insights for a better understanding of plasma skimming, they also highlight areas of limitations for further exploration. One limitation is that the optimization focused on the planar groove shape, leaving aspects like groove depth, sectional shape, and ridge-to-groove ratio unexplored. Future efforts might delve into these aspects for chasing better plasma skimming at the outer ridges with high hematocrit conditions. Furthermore, the current experimental RBP design is not suitable for hemolysis tests due to the small clearance between the axis and the mechanical bearings. Moreover, the study did not measure blood viscosity after altering hematocrit conditions, indicating a potential research opportunity to study the relationship between viscosity and plasma skimming.

## Conclusion

This study has designed an optimal SGB based on the revealed design strategy that aligns the groove shape with the direction of blood flow in the ridge gap, resulting in an excellent plasma skimming effect. With hematocrit conditions of up to 40%, the optimized SGB based on the design strategy was shown to have excellent plasma skimming efficiency. It also provided enough LCC to suspend the impeller in the axial direction within a specially designed experimental RBP.

## Data Availability

Due to privacy considerations, some data are not publicly available but can be accessed upon reasonable request to the authors.
